# Supplementary material for: Crawling and Gliding: A Computational Model for Shape-Driven Cell Migration
Source: PLoS Comput Biol. 2015 Oct 21;11(10):e1004280. doi: 10.1371/journal.pcbi.1004280 (PMC4619082; doi:10.1371/journal.pcbi.1004280)
Supplement: S1 Code — (ZIP) [file pcbi.1004280.s012.zip › release/tst/doc/html/structli.html]

Tissue Simulation Toolkit: li Struct Reference


Tissue Simulation Toolkit
 0.1.4.1


- Main Page
- Namespaces
- Classes
- Files

- Class List
- Class Hierarchy
- Class Members

Public Attributes |
List of all members

li Struct Reference

`#include <x11graph.h>`

|  |  |
| --- | --- |
| Public Attributes | |
| int | x1 |
|  | |
| int | y1 |
|  | |
| int | x2 |
|  | |
| int | y2 |
|  | |

## Member Data Documentation

int li::x1

Referenced by X11Graphics::CropSize().

int li::x2

Referenced by X11Graphics::CropSize().

int li::y1

Referenced by X11Graphics::CropSize().

int li::y2

Referenced by X11Graphics::CropSize().

---

The documentation for this struct was generated from the following file:

- x11graph.h


---

Generated on Thu Aug 14 2014 22:04:01 for Tissue Simulation Toolkit by  

 1.8.6
